# One-Step Thermal Treatment Route to Obtain ZnO Nanospheres from ZnAlEu LDH Nanotubes

**DOI:** 10.1021/acsomega.6c05773

**Published:** 2026-09-23

**Authors:** Thaina Calaça Futamata, Dimy Nanclares, Alexandre Candido Teixeira, Karina Torre da Fonseca, Danilo Mustafa

**Affiliations:** 28133 Instituto de Física da Universidade de São Paulo, São Paulo, São Paulo 05508-090, Brazil

## Abstract

This study presents a novel synthesis route for producing ZnO nanospheres via the controlled calcination of nanotubular layered double hydroxide (LDH) precursors. The thermal treatment promotes the transformation of the nanotubular LDH precursor into ZnO nanospheres through a restructuring process associated with LDH decomposition and oxide formation. Comprehensive characterization using XRD, SEM, HRTEM, and XRF mapping confirmed the formation of highly crystalline wurtzite ZnO-spheres. Although no distinct Eu_2_O_3_ reflections were initially observed by XRD, XRF mapping and XANES analyses revealed a heterogeneous distribution of europium within the calcined material. Eu-rich domains coexist with regions dominated by crystalline ZnO nanospheres, suggesting partial segregation of europium during the thermal transformation process. XANES analysis verified europium predominantly in the +3 oxidation state. The photocatalytic efficiency of the resulting material was evaluated through the degradation of Rhodamine 6G (R6G) under UV irradiation. The ZnO-spheres achieving ∼90% R6G degradation after 300 min, following pseudo-first-order kinetics (*k*_app_= 0.601 h^–1^). This significant activity is attributed to the synergistic effect of the spherical morphology, which provides a surface area and improved light-harvesting efficiency, and the synergistic role of the heterostructure formed between the ZnO-spheres and the Eu^3+^ rich domains, which facilitates spatial charge carrier separation at the interface, reducing electron–hole recombination. This work underscores the potential of LDHs as sacrificial templates for creating morphologically controlled mixed oxide photocatalysts and demonstrates the ZnO-spheres as a highly promising candidate for environmental remediation applications.

## Graphical abstract

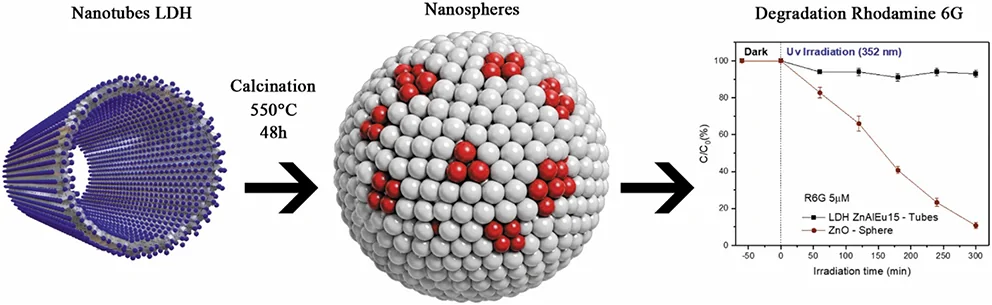

## 1 Introduction

Rhodamine 6G (R6G) is a xanthene-based dye widely employed as a model pollutant in photocatalytic degradation studies due to its well-defined molecular structure, intense visible absorption, and high photostability.^1^ It is frequently detected in wastewater from textile, printing, and chemical industries, posing significant environmental risks due to its persistence, potential toxicity, and low biodegradability.^2,3^ The aromatic rings and nitrogen-containing functional groups in its structure confer high resistance to biodegradation, underscoring the need for advanced oxidative treatment methods.^4,5^ Furthermore, its strong absorption band at approximately 526 nm enables straightforward monitoring of degradation kinetics via UV–Vis spectroscopy.^6^ These features make R6G an ideal probe molecule for assessing the efficiency of novel photocatalytic materials under controlled laboratory conditions.

Zinc oxide (ZnO) is a wide-bandgap semiconductor with a large exciton binding energy,^7^ long recognized for its versatility in applications ranging from electronic devices and sensors to photocatalysis.^8^ In particular, ZnO has attracted increasing attention for the degradation of persistent organic pollutants (POPs).^9^ For solar-driven applications, owing to its advantages over benchmark TiO_2_, including a broader absorption spectrum in the solar light, low cost, and nontoxicity.^10^ However, the photocatalytic performance of pristine ZnO is limited by its relatively wide bandgap, which requires high-energy photons that represent only a small fraction of the solar spectrum. One strategy to improve its efficiency involves increasing the surface area and tailoring its morphology to enhance light harvesting and charge separation efficiency.^11^

Layered double hydroxides (LDHs) are a class of anion-exchange materials with the general formula [M^II^_1–x_M^III^_*x*_(OH)^2^]^x+^[A_n–x/n_]^x–^·yH_2_O where M^2+^ and M^3+^ are divalent and trivalent metal cations, respectively, and A^n–^ is an interlayer anion.^12–15^ Isomorphic substitution of divalent cations in otherwise neutral brucite-like M­(OH)_2_ sheets with trivalent cations generates positive charges in the hydroxide layers, which are balanced by the incorporation of anions into the interlamellar space.^13,15,16^ Self-supported Zn/Al/Eu LDH nanotubes can be synthesized through a Pluronic P-123 worm-like micelle-based soft-templating route combined with the incorporation of Eu^3+^ ions into the LDH layers. This approach yields well-defined, multiwalled, hollow cylindrical nanotubes. These unique nanostructures, with an average internal diameter of ∼20 nm,^17–20^ serve as ideal sacrificial templates for generating novel oxide morphologies.

While numerous studies have reported on Eu doped ZnO in various morphologies, such as nanoparticles^21^ or thin films,^22^ the use of nanotubular LDHs as sacrificial precursors to engineer well-defined ZnO-spheres remains largely unexplored. Such a morphological transformation, from ordered nanotubular precursors to spherical architectures, is expected to provide enhanced light harvesting, higher surface-to-volume ratios, and improved dispersion of Eu species. These features highlight the potential of LDH-derived nanostructures as versatile platforms for designing advanced ZnO-based photocatalysts.

This work, therefore, introduces a novel morphology transformation route in which self-supported ZnAl with Eu^3+^ layered double hydroxide (LDH) nanotubes are converted into ZnO-spheres through controlled thermal decomposition. While LDHs have been widely explored as precursors for metal oxides, reports on the preservation of a well-defined, templated nanostructure during the transition from a tubular to a spherical morphology remain scarce. By combining a nanostructured soft-templating synthesis with calcination-induced restructuring, this study provides new insights into the relationship between precursor architecture and final oxide properties. The resulting nanospheres were thoroughly characterized by X-ray diffraction (XRD), thermogravimetric analysis (TGA), X-ray absorption near-edge structure (XANES), X-ray excited optical luminescence (XEOL), scanning and transmission electron microscopy (SEM/TEM), and their photocatalytic performance was assessed through the degradation of Rhodamine 6G under UV irradiation, serving as a proof-of-concept application to demonstrate the functional potential of the new nanosphere morphology. The findings offer valuable guidelines for tailoring ZnO-based nanostructures for environmental and optoelectronic applications. Rather than focusing on band gap optimization, the present study aims to establish a structure–morphology–function relationship by demonstrating how the thermal transformation of LDH nanotubes into ZnO nanospheres influences the photocatalytic response. The originality of this approach lies in the conversion of luminescent LDH nanotubes into mixed-oxide nanospheres while preserving complex hierarchical features inherited from the precursor architecture.

## 2 Materials and Methods

### 2.1 Materials

Zinc nitrate hexahydrate (Zn­(NO_3_)_2_·6H_2_O, 96%) and aluminum nitrate nonahydrate (Al­(NO_3_)_3_·9H_2_O, 98%) were obtained from LabSynth, while sodium hydroxide (NaOH, 97%) was supplied by Vetec. Europium­(III) nitrate hexahydrate (Eu­(NO_3_)_3_·6H_2_O) was synthesized in house by dissolving a stoichiometric amount of europium­(III) oxide (Eu_2_O_3_, 99%, CSTARM) in dilute nitric acid under reflux conditions, following a reported procedure.^23^ The organic anion benzene-1,3,5-tricarboxylic acid (BTC) and the triblock copolymer Pluronic P-123 were purchased from Sigma-Aldrich.

### 2.2 Synthesis

The sample, denoted as LDH ZnAlEu15-Tubes, with nominal composition [Zn_2_A_0.85_Eu_0.15_(OH)_6_]·(BTC^3–^)­0.33·pH_2_O, was synthesized via coprecipitation at constant pH under ambient pressure, following a modified procedure based on previous report.^17–20^ First, a micellar solution was prepared by dissolving Pluronic P-123 (0.15 wt %) and benzene-1,3,5-tricarboxylic acid (BTC, 11.5 mmol L^–1^) in 200 mL of deionized water. The pH was adjusted to 8 using NaOH. The solution was heated to 60 °C to ensure full dissolution of components and then cooled to room temperature. A second heating cycle at 60 °C was applied to promote micellization.

Subsequently, 10 mL of a 1 mol L^–1^ aqueous solution containing the metal nitrates Zn­(NO_3_)_2_·6H_2_O, Al­(NO_3_)_3_·9H_2_O, and Eu­(NO_3_)_3_·6H_2_O in a molar ratio of 2:0.85:0.15 was added dropwise (∼10 mL h^–1^) to the micellar solution under continuous stirring. The pH was maintained at 8 throughout the addition using a Metrohm 848 Titrino Plus automatic titrator. After complete addition, the resulting suspension was aged statically at 60 °C for 48 h. The solid product was collected by centrifugation, washed thoroughly with deionized water, and then subjected to two washing steps with methanol under sonication (15 min each) to ensure complete removal of the residual Pluronic P-123 template. Finally, the material was dried in an oven at 60 °C for 72 h.

### 2.3 Calcination

The as-synthesized LDH ZnAlEu15-Tubes was calcined in a static-air muffle furnace. The temperature was ramped from room temperature to 550 °C at a rate of 5 °C min^–1^ and held at the target temperature for 48 h.

Although the thermal decomposition of the LDH is completed at substantially lower temperatures, the extended calcination time was intentionally selected to ensure that the material reached a stable final morphology prior to structural, optical, and photocatalytic characterization. The objective of the present study was to investigate the properties of the fully transformed ZnO nanospheres rather than the kinetics of the morphology evolution.

### 2.4 Rhodamine 6G Photocatalytic Degradation

Rhodamine 6G (R6G, Sigma-Aldrich) was used as the model dye. A 5 μM aqueous solution was prepared at neutral pH (pH 6.5). In a typical experiment, 20 mL of the R6G solution was mixed with 20 mg of the catalyst (the ZnO-spheres derived from LDH calcination) and sonicated for 20 min to ensure uniform dispersion. Photocatalytic degradation was performed using a monochromatic excitation source at 352 nm provided by an FS5 spectrofluorometer (Edinburgh Instruments, UK) operating in continuous-output mode. The dye–catalyst suspension was kept in a sealed beaker and irradiated for 5 h. For reproducibility and comparative analysis, the photocatalytic experiments were independently repeated four times for both the ZnO nanospheres and the precursor LDH nanotubes under identical experimental conditions. The reported degradation curves correspond to the average values, and the error bars represent the standard error of the mean (SEM). Aliquots of 1.5 mL were withdrawn at regular intervals (0, 60, 120, 180, 240, and 300 min), centrifuged to remove the catalyst, and the absorbance of the supernatant was recorded at 526 nm to monitor degradation.

### 2.5 Kinetic Study of Photodegradation

The photodegradation kinetics of R6G were evaluated by fitting the experimental data to the pseudo-first-order kinetic model:

$$\ln\left(\frac{C}{C_{0}}\right)=k_{app}t$$

Where C_0_ and C are the initial and time-dependent concentrations of Rhodamine 6G, respectively, and *k*_app_ is the apparent rate constant. Linear fitting of ln­(C_0_/Ct) versus irradiation time was performed for both ZnO nanospheres and LDH nanotubes. The apparent rate constants obtained from four independent experiments were statistically compared using Welch’s *t* test with a significance level of *p* < 0.05.

### 2.6 Characterization

Powder X-ray diffraction (XRD) patterns were recorded in Bragg–Brentano geometry using a D8 Discover diffractometer (Bruker) with Cu Kα radiation (λ = 1.5418 Å, 40 kV, 30 mA) and a Lynxeye detector. Data were collected over a 2θ range of 4° to 70°, with a step size of 0.02°, an integration time of 1 s per step, and sample rotation at 15 rpm. Thermogravimetric analysis (TGA) was performed on a TGA Q500 instrument (TA Instruments) under an air flow of 60 mL·min^–1^, heating from room temperature to 800 °C at a rate of 5 °C·min^–1^. Transmission electron microscopy (TEM) images were obtained with a JEOL JEM-2100 microscope operated at 200 kV, equipped with a lanthanum hexaboride (LaB_6_) electron source, providing a spatial resolution of up to 0.2 nm. Scanning electron microscopy (SEM) analyses were performed using a JEOL JSM-7401F field emission scanning electron microscope (FESEM) operated at 5.0 kV with a magnification of 50,000×. Samples were gold coated prior to analysis. Photoluminescence excitation and emission spectra were recorded at room temperature using an FS5 spectrofluorometer (Edinburgh Instruments) equipped with a 150 W xenon arc lamp. Measurements were performed in solid-state using front-face geometry, with spectral correction applied to both excitation and emission channels. The synchrotron-based studies were conducted at the Carnaúba beamline^24^ from Sirius, the fourth generation synchrotron accelerator from the Brazilian Synchrotron Light Laboratory (LNLS) that is part of the Brazilian Center for Research in Energy and Materials (CNPEM), a private nonprofit under the Brazilian Ministry of Science, Technology and Innovations (MCTI). The studies were conducted at room temperature and pressure, at the Tarumã endstation, using a submicrometric X-ray beam. STXM and XRF images were recorded with excitation energy at 9725 eV, in which is found the highest beamline flux. The powder sample sustained in carbon tape was raster scanned in areas of 200 μm × 200 μm, with pixel size 2 μm, and 50 μm × 50 μm, with pixel size 0.5 μm. STXM signal was recorded using a Photodiode Alibava model AS04–105A, while XRF measurements were taken using two silicon drift detectors (SDD) model Vortex, 4 elements, from Hitachi. These images were analyzed using the software PyMCA.^25^ XANES and XEOL studies were performed simultaneously in energy scans around the Eu LIII and Zn K-edge. XANES signal was recorded in fluorescence mode using both the Vortex detectors, and the data analyzed using the software Athena from the suíte Demeter.^26^ XEOL total yield was acquired using a charge-coupled device (CCD) model QE-Pro-ABS (Ocean Optics), which covers the range from 200–950 nm. XEOL emission spectrum was acquired with the same detector at fixed energy of 7000 eV.

## 3 Results and Discussion

### 3.1 Structural Characterization

The diffraction pattern of the as-synthesized precursor (Figure 1) exhibits broad reflections typical of nanocrystalline layered double hydroxide (LDH) phase based on ZnAlEu (LDH ZnAlEu15-Tubes), with peaks matching the characteristic reflections previously reported for nanotubular LDHs intercalated with benzene-1,3,5-tricarboxylate (BTC) anions.^27^ No additional crystalline phases or impurities are detected, confirming the purity and structural integrity of the precursor prior to thermal treatment.

**Figure 1:**
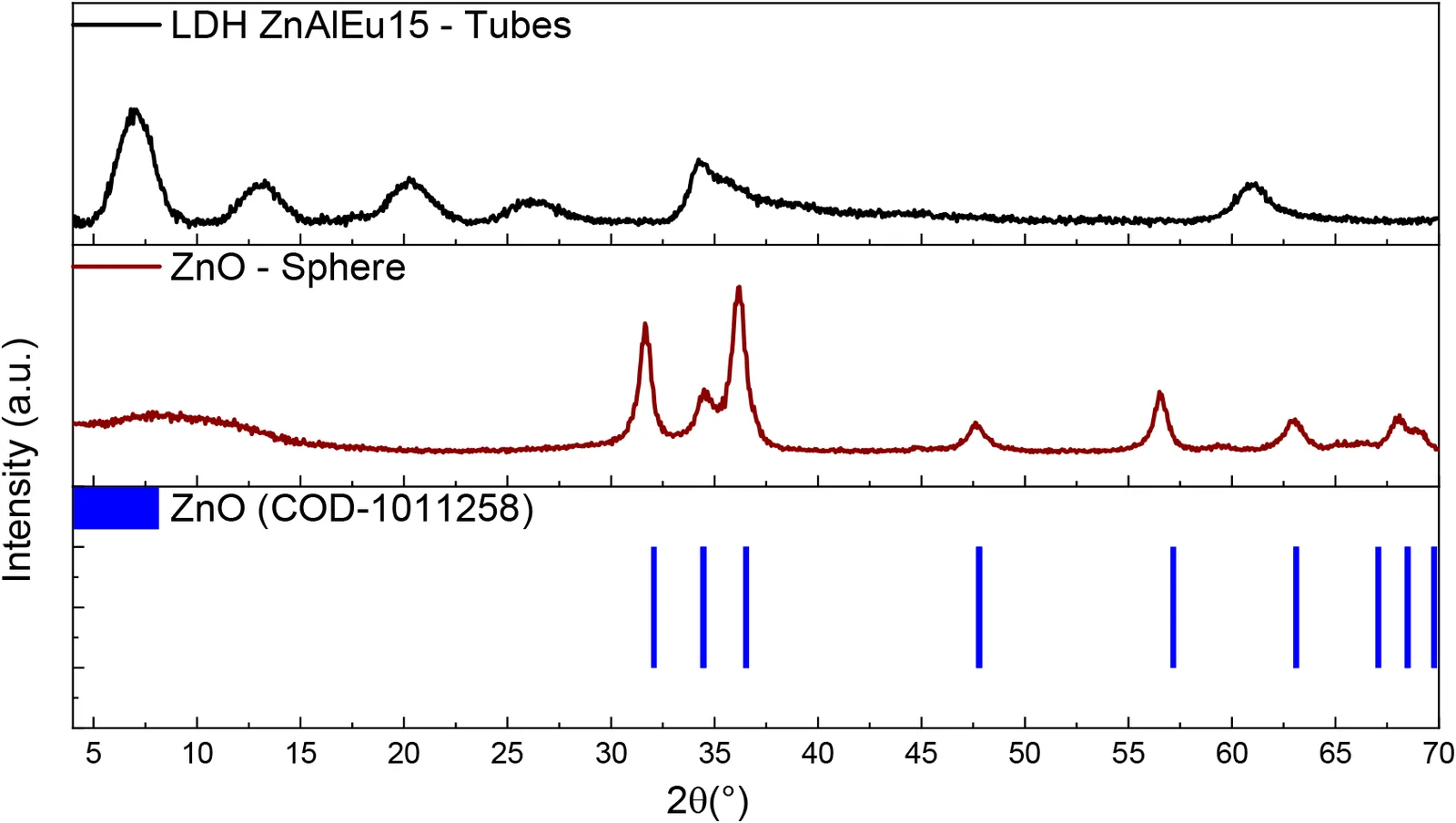
Powder X-ray diffraction patterns of the sample LDH ZnAlEu15-Tubes (black) in comparison with ZnO-spheres (brown) synthesis.

Upon calcination (resulting in the sample denoted as ZnO-spheres), the XRD profile undergoes a complete transformation. Reflections associated with the LDH phase disappear entirely, indicating complete phase transformation. The diffractogram displays the peaks marked in blue (2θ ≈ 31.8°, 34.7°, 36.2°, 46.8°, 56.7°, 61.7°, 65.9°, 68.2°; see Supporting Information, Table S1), are indexed to the (100), (002), (101), (102), (110), (103), (200), and (112) planes of hexagonal wurtzite ZnO, in agreement with Crystallographic Open Database (COD) card 1010339. The interplanar distances (d_hkl_) and lattice parameters (a = b = 0.323 nm, c = 0.514 nm) calculated from the pattern are consistent with literature values, confirming the presence of ZnO as a major phase.^28,29^

After calcination, the aluminum and europium initially present in the LDH layers are not detected as a separate crystalline phase. This behavior is consistent with previous reports on ZnAl LDHs, where the decomposition leads to the formation of ZnO nanocrystals homogeneously dispersed within an amorphous Al–O matrix, rather than to the crystallization of separate alumina phases.^30^ The absence of diffraction peaks related to europium oxides indicates that Eu^3+^ species are finely distributed in the amorphous Al–O matrix or partially incorporated into the ZnO lattice, in both cases at levels below the detection threshold of XRD.^31^

### 3.2 Thermal Transformation

Thermogravimetric analysis (TGA) of the LDH ZnAlEu15-Tubes precursor reveals three well-defined mass loss steps (Figure S1). The initial mass loss event, centered at ≈90 °C, attributed to the desorption of physisorbed water molecules from the external surface and interlayer galleries, held by weak hydrogen bonding.^32^

A second, more pronounced mass loss step occurs at ≈220 °C. This event is associated with (i) the removal of strongly coordinated interlayer water molecules, (ii) the dehydroxylation of the brucite-like layers themselves, and (iii) the condensation of hydroxyl groups.^8,33,34^ These processes collectively initiate the collapse of the characteristic layered LDH structure.^35^

The third and final mass loss step, observed between 300 and 620 °C, is assigned to the further dehydroxylation of the lattice, the progressive breakdown of the residual hydroxide structure, and the complete combustion and release of the intercalated BTC anions.^34,36^ The broad temperature range of this step is governed by the dense, poorly ordered packing of the collapsed layers, which kinetically hinders the diffusion and escape of the volatile decomposition products (CO_2_, H_2_O).^37^

The total mass loss recorded at 600 °C is in good agreement with the theoretical value calculated for the complete dehydroxylation and combustion of the LDH, confirming its full transformation into a mixed metal oxide. This oxide intermediate is the direct precursor for the formation of the final ZnO-spheres.

In summary, the TGA profile corroborates the successful formation of a thermally stable LDH structure and maps its stepwise decomposition pathway. The analysis confirms that a calcination temperature of 550 °C, as employed in this study, is sufficient to promote the extensive decomposition of the layered precursor and the formation of the mixed metal oxide phase. Minor remnants of the original lamellar framework may, however, persist locally due to kinetic stabilization during thermal treatment. This thermally induced transformation represents the fundamental step that enables the morphological evolution from the initial nanotubular architecture to the predominant nanospheric morphology.

### 3.3 Morphological Evolution

Scanning Electron Microscopy (SEM) analysis (Figure 2) reveals the coexistence of two distinct morphological features: (i) micrometric aggregates predominantly composed of highly crystalline ZnO nanospheres and (ii) residual plate-like structures inherited from the original LDH precursor. Although the material exhibits intrinsic morphological heterogeneity after calcination, the spherical nanoparticle aggregates clearly constitute the predominant morphology observed throughout the analyzed regions, while the lamellar domains remain restricted to localized residual areas. Although these lamellar-like structures preserve aspects of the precursor morphology, the absence of characteristic LDH reflections in the XRD pattern indicates that the original layered crystallographic order was lost during calcination. This observation indicates that, although the thermal treatment largely decomposed the lamellar precursor, localized regions of the initial morphology persisted, likely stabilized by slower diffusion of decomposition gases or local compositional heterogeneity.

**Figure 2:**
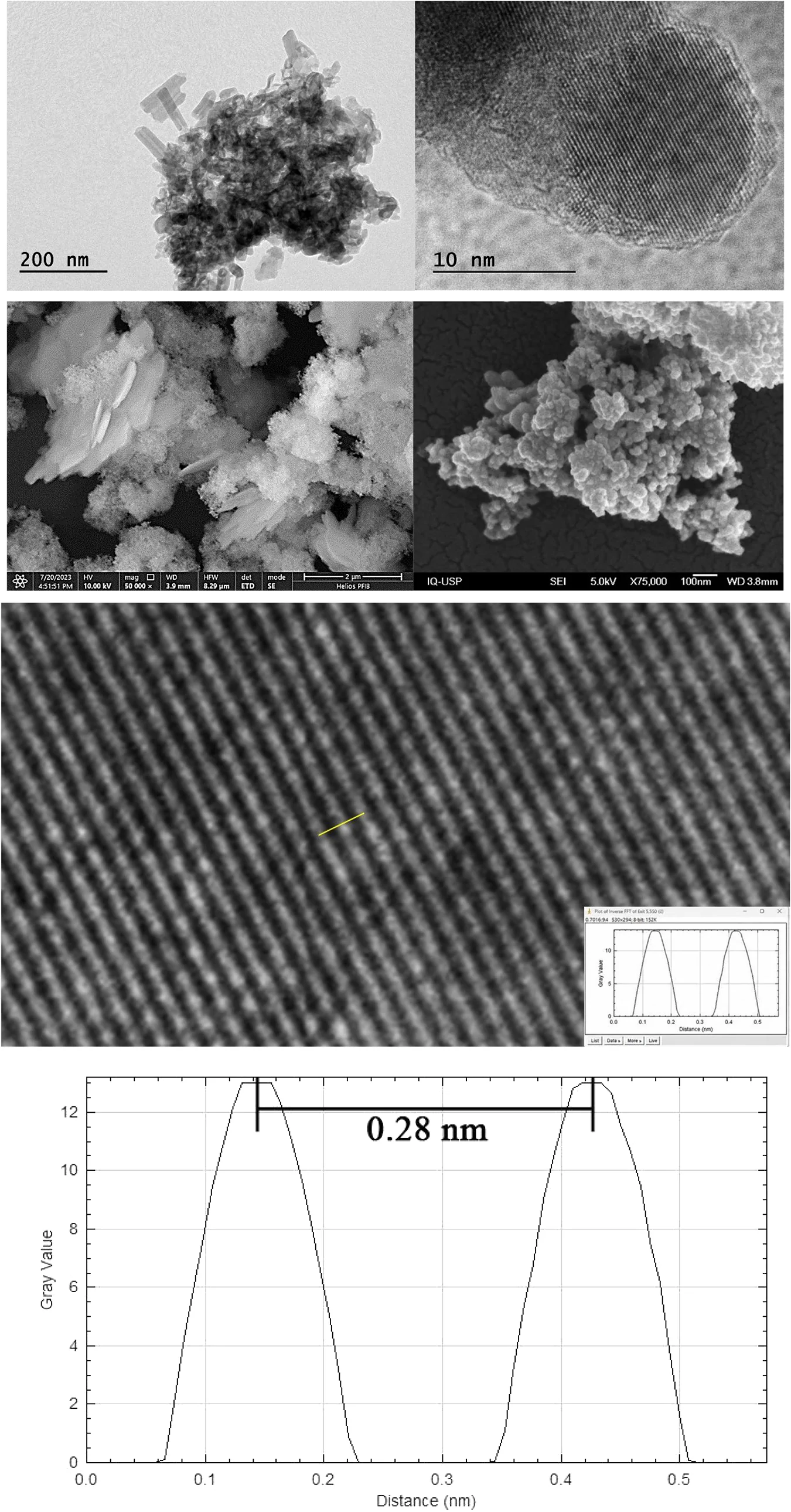
Above: on the left, transmission electron microscopy of the sample LDH ZnAlEu15-Tubes before the thermal treatment showing the existence of both plates and nanotubes. On the right, the same sample after the 550 °C treatment. Below: Scanning Electron Microscopy (SEM) of ZnO-sphere showing LDHs in micrometric size and agglomerates of ZnO-spheres. Digital image processing and Fast Fourier Transform (FFT) analyses were performed using ImageJ software (National Institutes of Health, USA) to determine the interplanar spacing from the HRTEM micrographs.

Crucially, high-resolution TEM (HRTEM) of individual nanospheres reveals well-resolved lattice fringes with a dominant interplanar spacing of 0.28 ± 0.01 nm, obtained from FFT analysis calibrated by the 10 nm scale bar. This value matches the (100) planes of wurtzite ZnO (d_100_ = 0.279 nm), and the corresponding reciprocal-space spots appear as a sharp pair aligned close to the horizontal axis, consistent with a nearly single-crystalline domain. The Fast Fourier Transform (FFT) pattern derived from the HRTEM image (Figure 2) further confirms the high structural quality of the nanoparticle, with reflections readily indexed to the wurtzite structure of ZnO, in full agreement with the XRD results.

X-ray Fluorescence (XRF) mapping elucidates the chemical distribution of Eu and Zn within the ZnO-spheres sample (Figure 3a–c). Maps were generated for the (a) Eu L-alpha and (b) Zn K-alpha emission lines, with a composite image (c) showing their correlative distribution. The analysis reveals a clear chemical segregation: intense Eu signals (red) are highly localized within large, massive microparticles, consistent with residual LDH phases. In stark contrast, the Zn signal (blue) is strong and ubiquitously distributed throughout the sample, aligning with the abundant nanoparticle agglomerates observed by SEM and TEM. The composite image confirms the prevalence of the Zn signal and shows that the intense Eu hotspots are confined to specific micron-sized domains.

**Figure 3:**
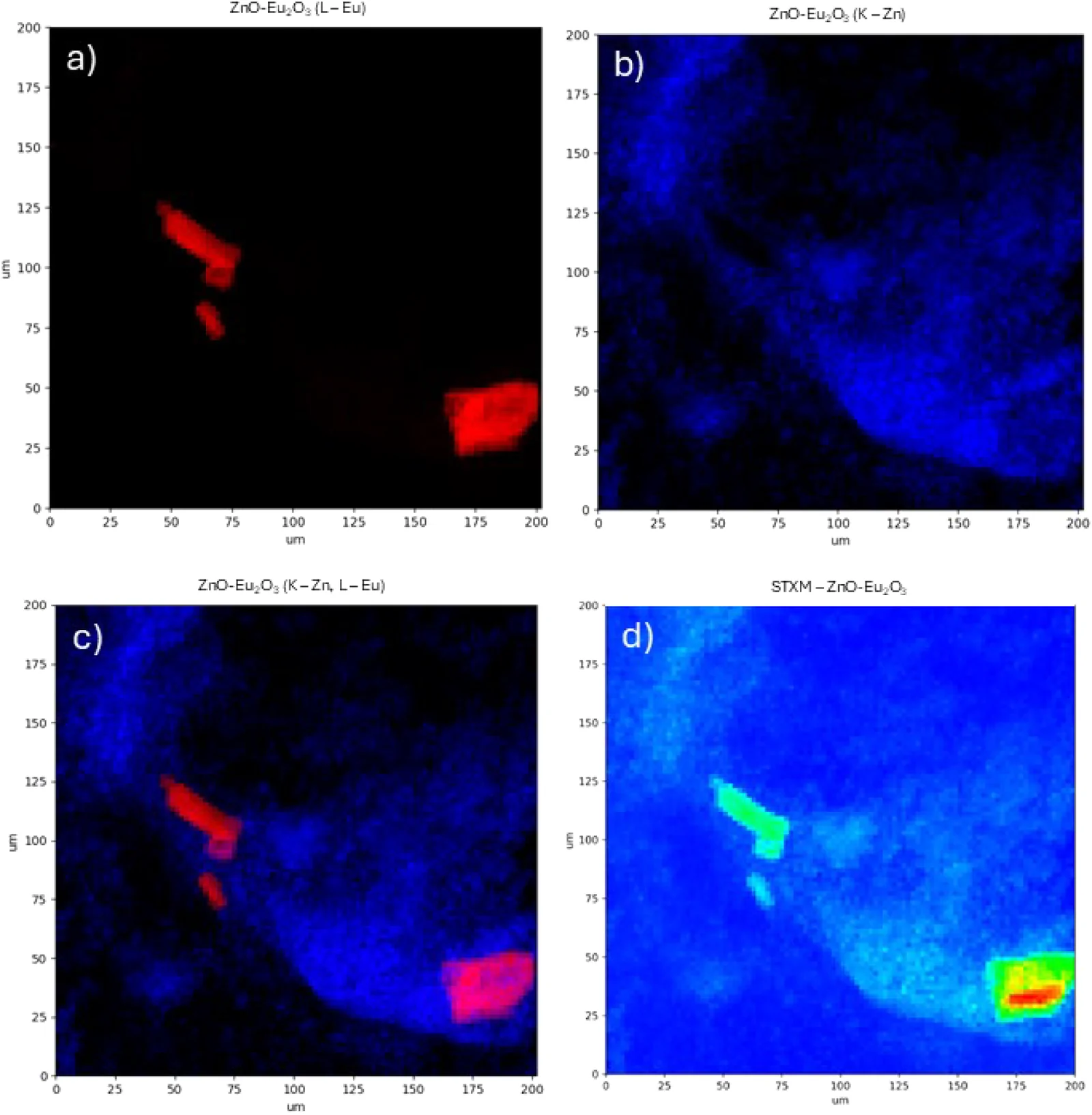
(a–c) XRF mapping of the elemental distribution in the ZnO-sphere sample: (a) Eu L-alpha emission, (b) Zn K-alpha emission, and (c) composite image overlay of Eu (red) and Zn (blue) signals. The maps (200 μm × 200 μm, pixel size: 2 μm) reveal a clear chemical segregation, with Eu-rich domains localized in specific microparticles and Zn signal ubiquitously distributed throughout the nanoparticle agglomerates. (d) STXM image of the ZnO-sphere sample recorded at an excitation energy of 9725 eV using absorption contrast. The images show mesoscale density at magnifications 200 μm × 200 μm (pixel size: 2 μm). Warmer colors (red) indicate regions of higher X-ray absorption (denser/thicker agglomerates), while cooler colors (blue-green) indicate regions of lower mass thickness.

The spatial resolution of the XRF maps (2 μm pixel size) exceeds the dimensions of individual ZnO nanospheres by more than 2 orders of magnitude, and therefore the elemental maps primarily reflect mesoscale chemical heterogeneities between agglomerated domains rather than atomic-scale Eu distribution within the ZnO lattice. To address the question of Eu incorporation at the local structural level, the optical spectroscopy data provide complementary and more sensitive information. The dominance of the ^5^D_0_ → ^7^F_2_ electric dipole transition over the ^5^D_0_ → ^7^F_1_ magnetic dipole transition in both the XEOL and PL emission spectra (Figures 4d and S3) is a well-established indicator that Eu^3+^ ions occupy sites devoid of inversion symmetry. This observation is consistent with partial incorporation of Eu^3+^ into the wurtzite ZnO lattice (space group *P*6_3_mc, noncentrosymmetric), where Zn^2+^ sites lack a center of inversion, although incorporation into an amorphous Al–O or Eu-oxide matrix with similarly low local symmetry cannot be excluded. The absence of detectable Eu_2_O_3_ reflections in the XRD pattern further indicates that any Eu-rich domains formed during calcination are either amorphous, nanodispersed below the XRD coherence length, or present in quantities below the detection threshold. Collectively, XRD, XANES, XEOL, and PL data converge to a picture in which europium is distributed across two environments: a fraction segregated into amorphous or nanocrystalline Eu-rich clusters visible as mesoscale heterogeneities in XRF maps, and a fraction incorporated into the ZnO lattice at the atomic scale, the latter evidenced by the noncentrosymmetric optical signature of the Eu^3+^ emitter.

**Figure 4:**
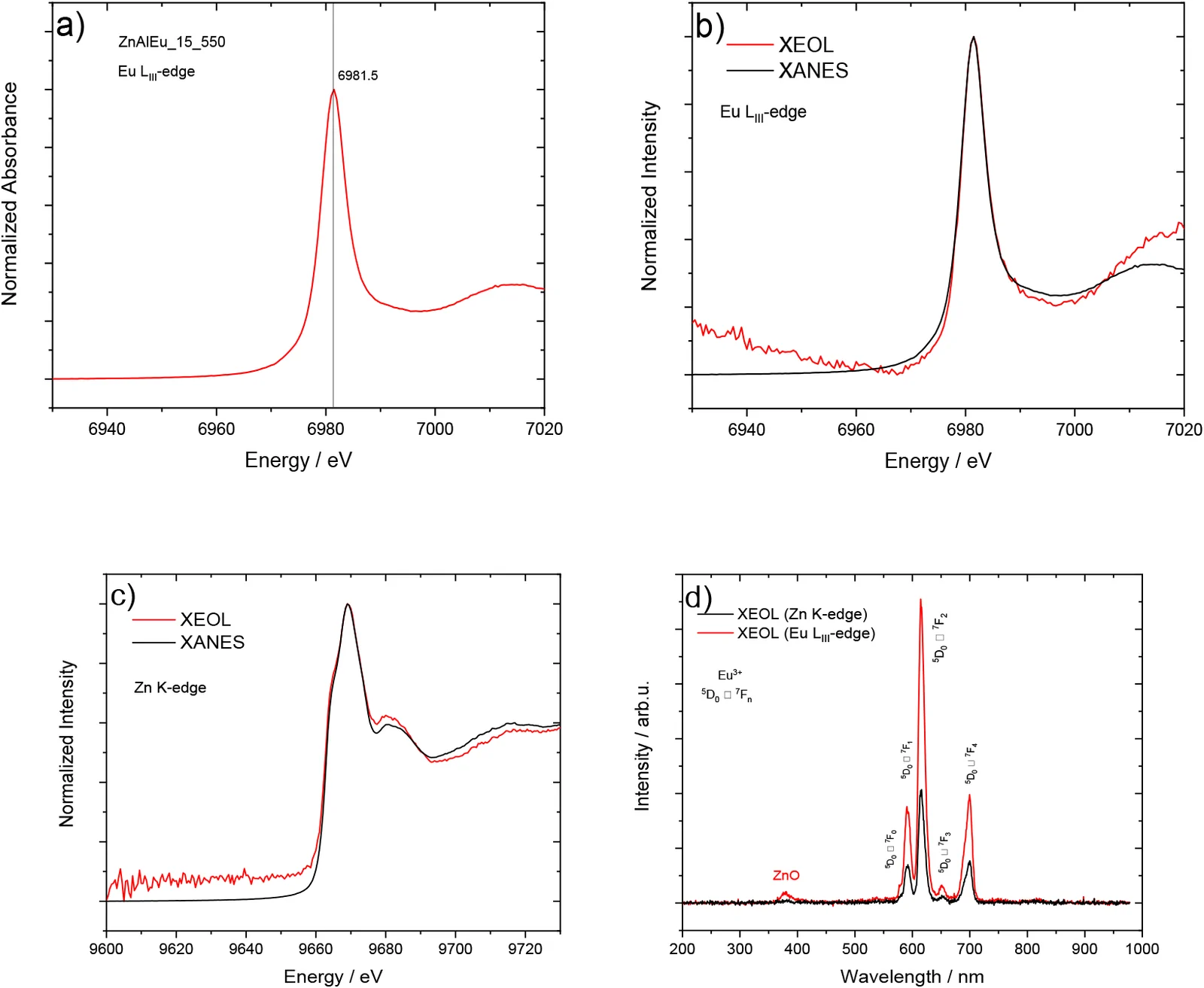
(a) X-ray Absorption Near Edge Structure (XANES) spectrum at the Eu LIII-edge of the ZnO-spheres composite, confirming the predominance of Eu^3+^ species. (b) Combined XANES/XEOL yield spectrum showing the correlation between X-ray absorption and optical emission intensity. (c) XEOL excitation profiles collected at the Eu LIII-edge and Zn K-edge, indicating efficient radiative relaxation pathways upon core-level excitation. (d) Deconvoluted XEOL emission spectrum under 7000 eV excitation, displaying the characteristic sharp ^5^D_0_ → ^7^F_n_ (*n* = 0–4) transitions of Eu^3+^ in the orange-red region, along with a broad near-band-edge emission band centered at ∼380 nm attributed to the ZnO host matrix, evidencing the dual luminescent contribution of dopant and semiconductor phases.

Scanning Transmission X-ray Microscopy (STXM) was employed to investigate mesoscale density variations (Figure 3d). Images acquired at an excitation energy of 9725 eV using absorption contrast show a heterogeneous material distribution. Warmer-colored regions (red) correspond to areas of higher X-ray absorption, indicating denser and/or thicker material agglomerates. Cooler-colored regions (light blue to green) indicate areas of lower mass thickness. This distribution is consistent with the SEM and TEM observations, where the dense agglomerates correlate with the ZnO-spheres and the less dense regions with the residual LDH platelets or thinner areas.

The local electronic structure and luminescence properties of the ZnO-spheres composite were probed by X-ray absorption and luminescence spectroscopy. X-ray Absorption Near Edge Structure (XANES) at the Eu L_III_-edge was employed to determine the oxidation state of europium, a critical parameter for understanding the optical properties of the micro- and nanoparticles present in the composite. The XANES spectrum (Figure SI-2a) is consistent with the predominance of Eu^3+^ species, as evidenced by the intense white-line feature and its energy position, which closely resembles previously reported spectra of Eu_2_O_3_ and Eu^3+^ -containing compounds available in the literature.^38^

The luminescence response was investigated via X-ray Excited Optical Luminescence (XEOL) measurements, conducted simultaneously with XANES. The XANES/XEOL yield spectrum (Figure 4b) shows that the correlation between XEOL intensity and X-ray absorption suggests that a significant fraction of the absorbed energy is released via radiative decay channels upon excitation at both the Eu L_III_-edge and the Zn K-edge (Figure 4c).

The deconvoluted XEOL emission spectrum, obtained under 7000 eV excitation (Figure 4d), displays the characteristic sharp-line transitions of Eu^3+^, ascribed to the ^5^D_0_ → ^7^F_n_ (n = 0, 1, 2, 3, 4) transitions in the orange-red region. A broader emission band centered at approximately 380 nm is also observed and attributed to the near-band-edge emission of ZnO.^39,40^ This confirms the dual nature of the composite’s luminescence, originating from both the Eu^3+^ dopant ions and the ZnO host matrix.

Additional photoluminescence (PL) measurements were performed to further investigate the optical response of the ZnO nanospheres (Figure S2). The excitation and emission spectra display the characteristic transitions of Eu^3+^ ions, confirming the preservation of the luminescent properties after thermal transformation of the LDH precursor. The emission spectrum is dominated by the ^5^D_0_ → ^7^F_2_ transition in the red spectral region, indicative of Eu^3+^ ions located in noncentrosymmetric environments. These results are fully consistent with the XEOL measurements and further corroborate the coexistence of luminescent Eu^3+^ species within the ZnO composite system.

In summary, the multitechnique characterization encompassing SEM, HRTEM, STXM, XRF, XANES, and XEOL provides a comprehensive portrait of the composite material. Electron microscopy (SEM/HRTEM) revealed the core morphology of crystalline ZnO-spheres coexisting with residual LDH microparticles, a heterogeneity corroborated by STXM and XRF mapping, which highlighted the correlation between dense Zn-rich agglomerates and segregated, Eu-rich domains. The XANES analysis definitively established the trivalent state of europium (Eu^3+^), while XEOL spectroscopy uncovered the efficient radiative relaxation of the system and its dual luminescence origin: sharp lines from Eu^3+^ emitters and a broad band from the ZnO matrix. Collectively, these findings confirm the efficacy of the reported one-step thermal treatment of luminescent LDH nanotubes. Despite the heterogeneous precursor, the process successfully yields well-defined ZnO-spheres with high crystallinity. The method produces a complex composite system where these highly crystalline nanospheres coexist with residual LDH-derived microparticles, resulting in functional morphologies and attractive optical properties for both the ZnO matrix and the Eu^3+^ ions. These findings demonstrate that the reported thermal transformation is not merely a phase conversion process, but rather a morphology-driven restructuring route capable of generating multifunctional oxide nanostructures with coupled structural, optical, and photocatalytic properties.

These findings further demonstrate that the ZnAlEu LDH nanotubes function as sacrificial templates, in which the precursor architecture actively guides the structural reorganization occurring during calcination. In addition to supplying the constituent metal species, the nanotubular framework guides the formation of a distinct ZnO nanosphere morphology, illustrating how the structural characteristics of the precursor can be exploited to engineer oxide materials with tailored morphologies.

### 3.4 Rhodamine Degradation

The photocatalytic performance of ZnO-spheres was evaluated through the degradation of Rhodamine 6G (R6G) under UV irradiation at 352 nm. Figure S3 shows the time-dependent UV–vis absorption spectra of R6G in the presence of the catalyst. The characteristic absorption band of R6G gradually decreases with increasing irradiation time, indicating progressive degradation of the dye. After 300 min of irradiation, the intensity of the main absorption band decreased by approximately 90%, indicating the extensive degradation of Rhodamine 6G. Control experiments performed under dark conditions for 60 min showed negligible degradation, confirming that the observed activity originates from the photocatalytic process mediated by ZnO-spheres. To evaluate the influence of morphology on the photocatalytic response, identical experiments were also conducted using the precursor LDH nanotubes. All measurements were repeated four times to ensure reproducibility and statistical consistency.

The temporal evolution of the relative concentration C_0_/C for both ZnO nanospheres and precursor LDH nanotubes is shown in Figure 5a. The nanospheres exhibited significantly enhanced photocatalytic activity, achieving approximately 90% degradation after 300 min of UV irradiation, whereas the nanotubular precursor displayed only minor degradation under the same conditions. The degradation curves represent the average of four independent experiments, with error bars corresponding to the standard error of the mean (SEM).

**Figure 5:**
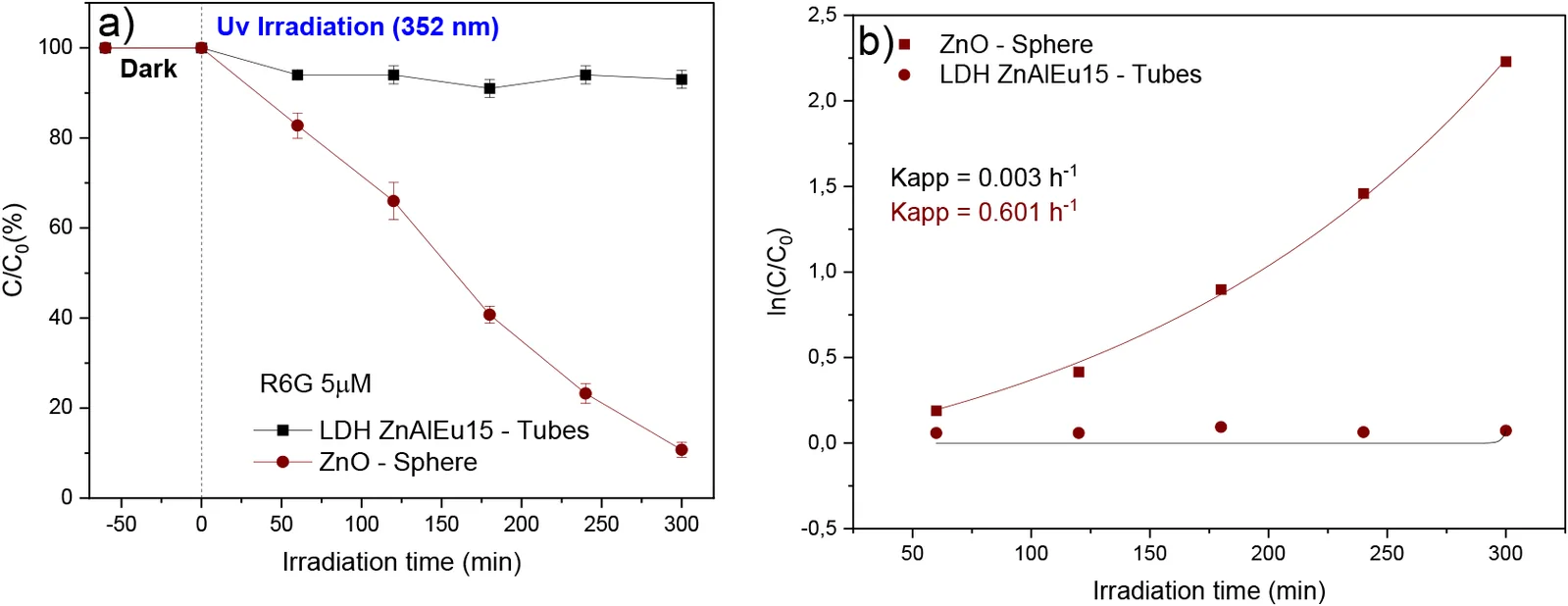
Comparison between the photocatalytic degradation performances of LDH-derived ZnO nanospheres and precursor LDH nanotubes under UV irradiation. (a) Temporal evolution of the relative Rhodamine 6G concentration (C_0_/C) as a function of irradiation time. Data correspond to the average of four independent experiments, and error bars represent the standard error of the mean (SEM). (b) Pseudo-first-order kinetic plots ln­(C_0_/C) versus irradiation time for both morphologies. The nanospheres exhibit a substantially higher apparent rate constant, confirming the decisive role of morphology transformation in enhancing photocatalytic activity.

The markedly superior performance of the nanospheres demonstrates that the thermal transformation from nanotubular LDH precursors into ZnO spherical oxide structures plays a decisive role in enhancing photocatalytic efficiency. This improvement can be attributed to the increased accessible surface area, enhanced light scattering associated with the spherical morphology.

As shown in Figure 5b, the plot of ln­(C/C_0_) versus irradiation time, confirms that the degradation follows pseudo-first-order kinetics. The calculated rate constant for the ZnO-spheres is *k*_app_ = 0.601 h^–1^.

This kinetic performance demonstrates the beneficial role of morphology transformation and Eu incorporation in improving photocatalytic activity,^4^ underscoring the beneficial role of the unique nanosphere morphology and europium incorporation. Although the absolute degradation efficiency is moderate, these results are significant as a proof of concept, confirming that the LDH derived ZnO-spheres are catalytically active. The study primarily highlights the role of morphology transformation rather than the optimization of photocatalytic performance. The statistically significant enhancement observed after calcination demonstrates that the transformation from nanotubular LDH precursors into ZnO nanospheres plays a decisive role in promoting photocatalytic activity.

Under UV excitation, ZnO absorbs photons and generates electron–hole pairs. In the absence of dopants, rapid recombination of these charge carriers typically limits photocatalytic efficiency. The enhanced photocatalytic efficiency can be rationalized by the presence of Eu-rich domains distributed within the ZnO-based composite, which may act as electron trapping sites and promote charge separation (Figure 3).

Under UV excitation, the ZnO semiconductor generates electron–hole pairs. Due to the band alignment at the interface between these distinct phases (ZnO and the Eu-rich residual domains), the photogenerated charge carriers can be spatially separated. A plausible mechanism involves the transfer of photogenerated electrons from the ZnO conduction band toward interfacial Eu-rich domains. Whether this process occurs through direct electron trapping by Eu containing species or is mediated by interface states and structural defects cannot be determined from the present results. Nevertheless, the heterogeneous distribution of Eu-rich domains suggests that these regions may act as charge-separation centers, suppressing electron–hole recombination and facilitating the generation of reactive oxygen species.

This process inhibits direct recombination and leads to a more efficient charge separation process. The trapped electrons can subsequently transfer to molecular oxygen adsorbed on the catalyst surface, producing superoxide radicals (•O_2_^–^), while the photogenerated holes oxidize surface hydroxyl groups or water molecules to yield hydroxyl radicals (•OH).^6^ Both reactive oxygen species play a decisive role in the degradation of R6G by attacking the xanthene ring and breaking down the conjugated chromophore system. Thus, the synergistic effect of enhanced charge separation and the increased generation of •OH and •O_2_^–^ radicals explains the superior photocatalytic performance of ZnO-spheres compared to undoped ZnO.

Among the possible photocatalytic pathways, the most plausible mechanism involves a continuous electron transfer process until the Fermi level of the ZnO composite shifts toward a more negative potential. This band alignment promotes the transfer of photogenerated electrons to the Eu-rich phase, from which they can be transferred to adsorbed oxygen species, enhancing the formation of •O_2_^–^ radicals. Simultaneously, photogenerated holes in the valence band of ZnO promote the production of hydroxyl radicals (•OH) from surface hydroxyl groups and water molecules.^41^ These •OH species are recognized as the dominant oxidative agents responsible for the cleavage of the xanthene ring in R6G, ultimately driving its mineralization.

Overall, these findings demonstrate that the ZnO-spheres derived from LDH precursors combine morphological and electronic advantages that act synergistically to enhance photocatalytic efficiency. The hollow spherical architecture promotes effective light harvesting and provides a high density of active surface sites, while the formation of a heterostructure with the Eu^3+^ rich domains promotes efficient interfacial charge separation, which modulates carrier lifetimes, hinders recombination processes, and facilitates the generation of reactive oxygen species. The calculated apparent rate constant of 0.601 h^–1^ and the 90% degradation efficiency confirm a significantly improved activity compared to many pure ZnO systems, highlighting its potential as a promising photocatalyst for the degradation of persistent organic pollutants.

## 4 Conclusion

This work successfully demonstrates a novel and effective strategy for the synthesis of heterostructured ZnO-spheres through the controlled thermal decomposition of specially designed ZnAlEu-LDH nanotube precursors. The key achievement lies in the morphological transformation from one-dimensional nanotubes to three-dimensional nanospheres, a process driven by the decomposition and recrystallization of the LDH framework.

The multitechnique characterization encompassing XRD, SEM, HRTEM, STXM, XRF, XANES, and XEOL provided a comprehensive portrait of the resulting composite. It confirmed the predominant formation of highly crystalline wurtzite ZnO-spheres, as evidenced by well-defined lattice fringes and sharp FFT patterns in HRTEM, coexisting with minor Eu-rich residual domains derived from the LDH precursor. This chemical heterogeneity and the stable trivalent state of europium (Eu^3+^) were further elucidated by XRF mapping and XANES, respectively. Furthermore, the material exhibited a dual luminescence mechanism, emitting from both the ZnO matrix and the Eu^3+^ ions, a property uncovered by XEOL spectroscopy.

As a proof of concept, the photocatalytic performance was evaluated through the degradation of Rhodamine 6G. The ZnO-spheres demonstrated significantly enhanced photocatalytic activity compared to the precursor LDH nanotubes, achieving approximately 90% degradation of Rhodamine 6G under UV irradiation after 300 min. Kinetic analysis revealed pseudo-first-order behavior with an apparent rate constant of 0.601 h^–1^, compared with 0.003 h^–1^ for the precursor LDH nanotubes under identical experimental conditions.

In summary, this study provides valuable insights into the relationship between precursor morphology and the final properties of mixed metal oxides. The presented synthesis route is a versatile platform for designing advanced photocatalysts. The ZnO-spheres emerge as a highly promising material for environmental remediation applications, combining the benefits of a surface area morphology with improved charge separation efficiency. Future work will focus on optimizing the Eu loading and evaluating the catalyst’s performance under visible light to assess its practical applicability. Therefore, the photocatalytic experiments validate the concept that the nanosphere morphology obtained from LDH precursors is functional for pollutant degradation. This establishes a basis for future optimization and application of such nanostructures in environmental catalysis.

## Supplementary Material



## Notes

Thermogravimetric analysis; X-ray diffraction data; ZnO-sphere structural parameters; photoluminescence spectra; UV–Vis absorption spectra during Rhodamine 6G degradation; and a proposed photocatalytic mechanism (PDF)
