# Supplementary material for: One-Step Thermal Treatment Route to Obtain ZnO Nanospheres from ZnAlEu LDH Nanotubes
Source: ACS Omega. 2026 Sep 23;11(39):59512–21. doi: 10.1021/acsomega.6c05773 (PMC13648062; doi:10.1021/acsomega.6c05773)
Supplement: Supplementary file 1 [file ao6c05773_si_001.pdf]

Supporting Information

One-step thermal treatment route to obtain ZnO nanospheres from ZnAlEu LDH nanotubes

*Thaina Calaça Futamata, Dimy Nanclares, Alexandre Candido Teixeira, Karina Torre da Fonseca and Danilo Mustafa**

Instituto de física da Universidade de São Paulo, 05508-090 São Paulo, SP, Brazil:

***Email: dmustafa@usp.br**

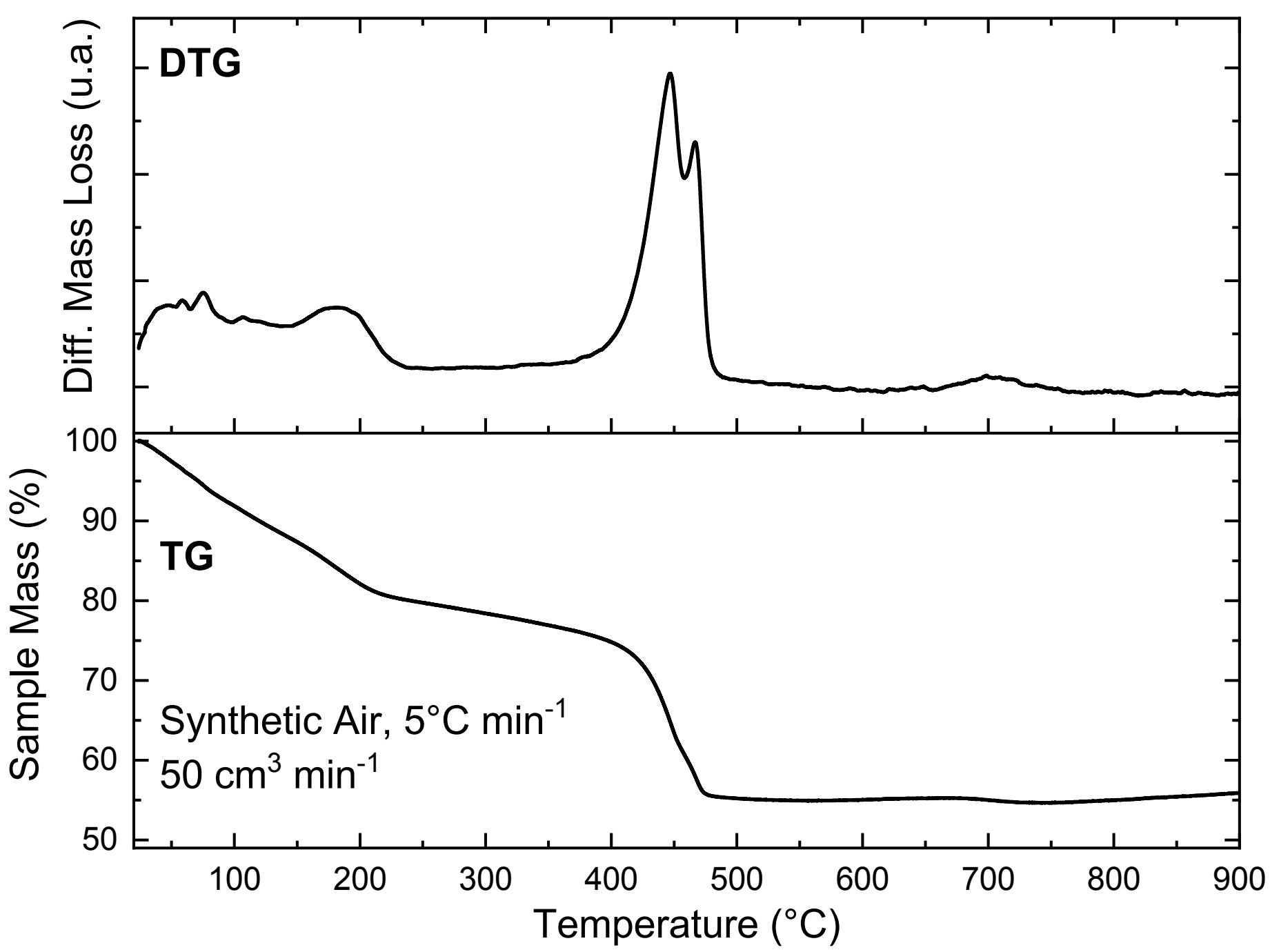

Figure S1. Thermogravimetric analysis (TGA) curve of the LDH ZnAlEu15–Tubes precursor, displaying three main mass loss steps: (i) removal of physisorbed and interlayer water (~90 ˚C); (ii) dehydroxylation of the layers and structural collapse (~220 ˚C); and (iii) further dehydroxylation and combustion of BTC anions (300–620 ˚C). The total mass loss at 600 ˚C confirms the transformation of the LDH into the mixed metal oxide precursor of the ZnO-spheres.

Table S1 – Comparison between experimental XRD data and standard ZnO reference pattern

**(hkl) planes**	2θ (°)		Intensity		O_{hkl}		d_{hkl}	
	JCPDS	Sphere	JCPDS	Sphere	JCPDS	Sphere	JCPDS	Sphere
**(100)**	32.070	31,973	51	76	0,24	0,28	2.81	2.81
**(002)**	34.467	34,881	39	38	0,17	0,14	2.60	2.59
**(101)**	36.533	36,435	100	100	0,41	0,37	2.47	2.47
**(102)**	47.790	47,012	23	17	0,08	0,06	1.91	1.92
**(110)**	57.168	56,838	35	36	0,10	0,13	1.62	1.62

Table S2 – ZnO-sphere properties

**(hkl) planes**	Crystallite size (nm)	Strain (10^{-3})	Bond length (A)	Lattice volume (A)
**(100)**	9.27	0.78	1.98	47.47
**(002)**	21.52	0.33		
**(101)**	9.78	0.65		
**(102)**	5.79	0.85		
**(110)**	8.54	0.49		

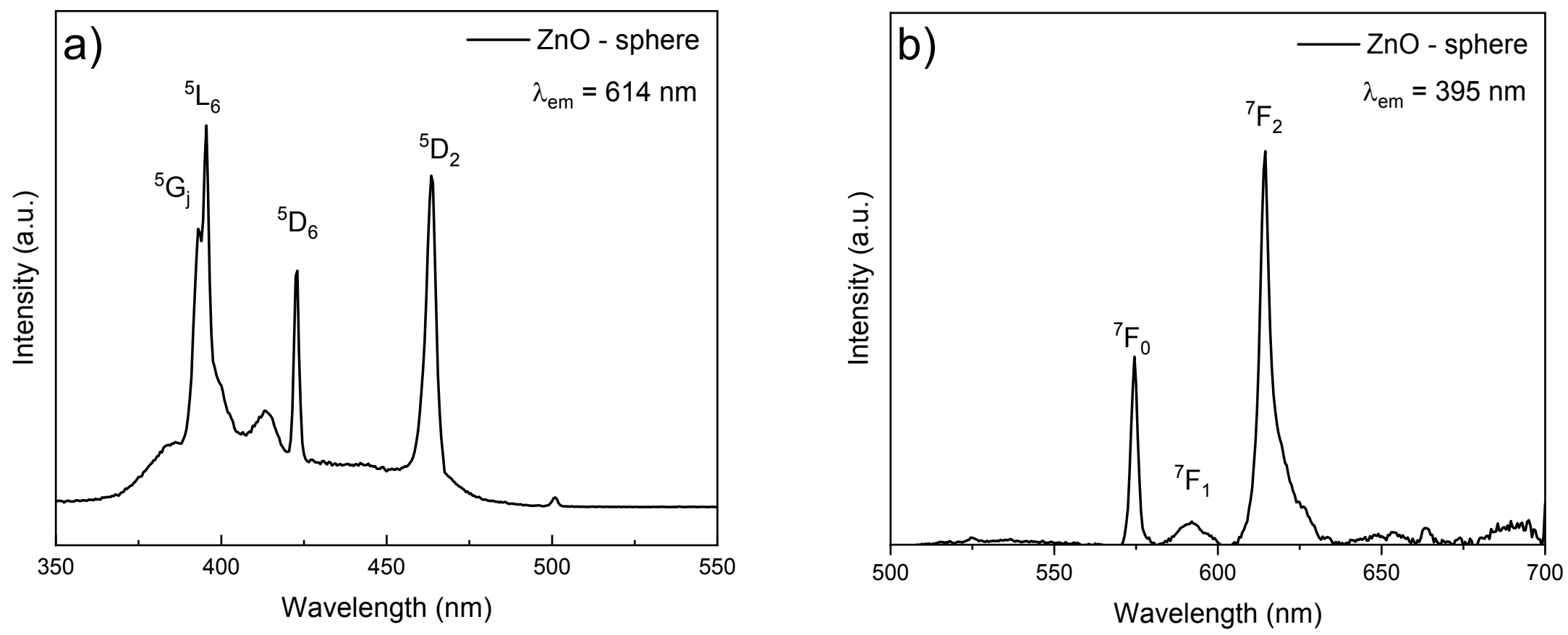

Figure S2. Photoluminescence excitation (a) and emission (b) spectra of ZnO nanospheres recorded at room temperature. The spectra display the characteristic Eu^{3+} intra-4f transitions, confirming the preservation of the luminescent properties after thermal transformation of the LDH precursor.

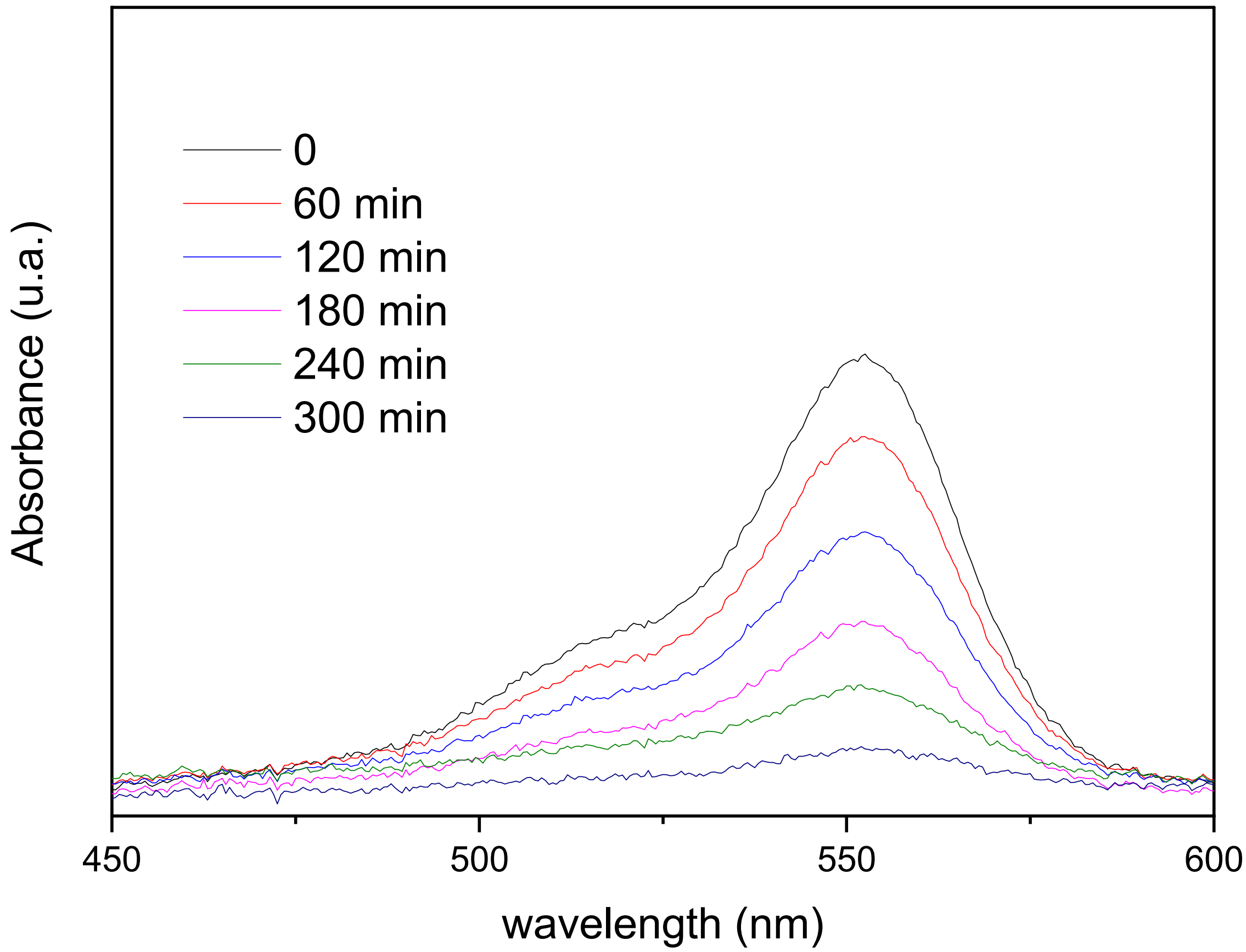

Figure S3. Time-dependent UV−Vis absorption spectra of Rhodamine 6G (R6G) during photocatalytic degradation under UV irradiation (352 nm) in the presence of ZnO-sphere

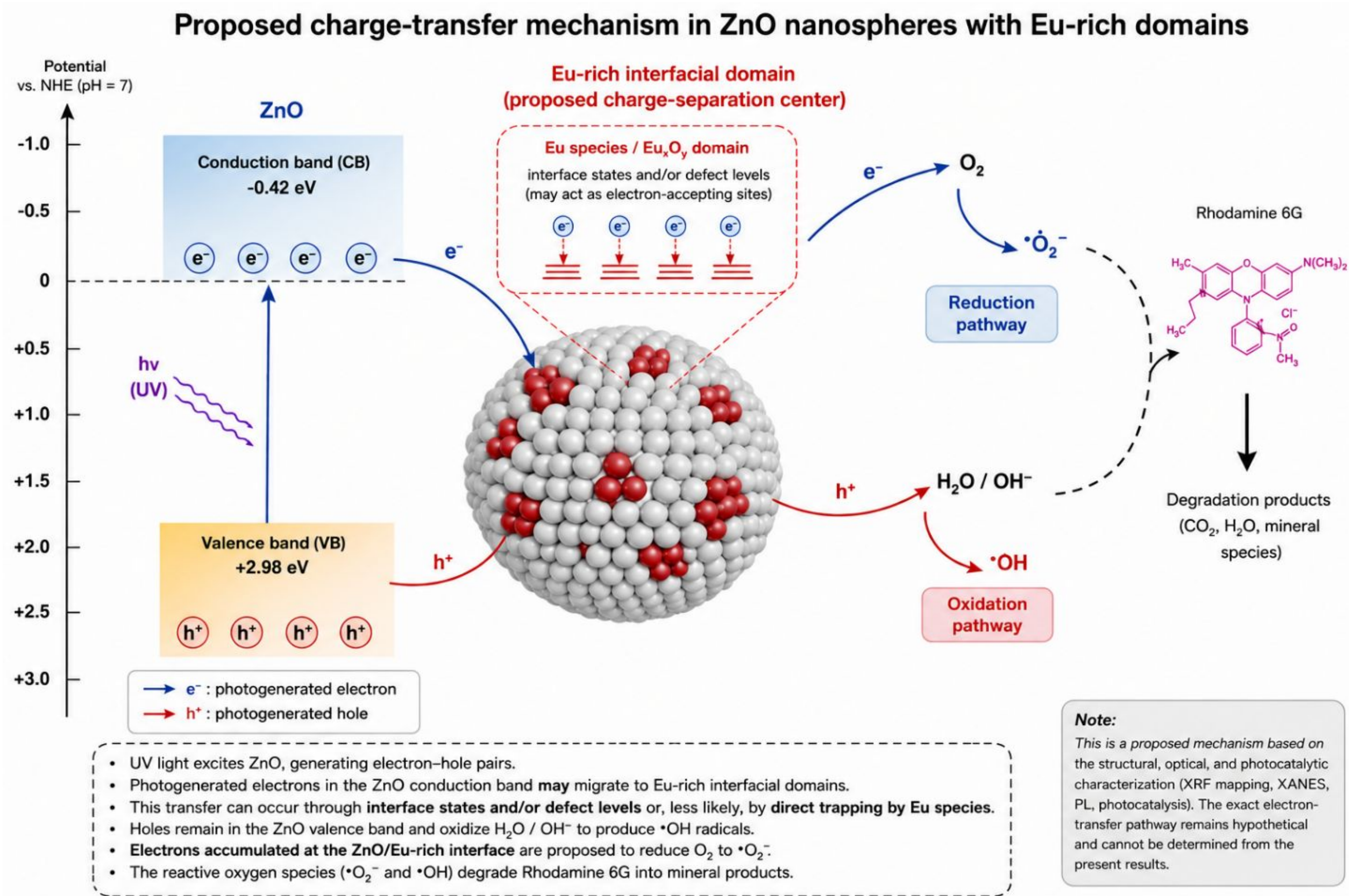

Figure S4. The diagram represents a plausible mechanism based on the structural, optical and photocatalytic characterization; the exact electron-transfer pathway has not been experimentally verified.
